# Oxidative stress and mitochondrial responses to stress exposure suggest that king penguins are naturally equipped to resist stress

**DOI:** 10.1038/s41598-019-44990-x

**Published:** 2019-06-12

**Authors:** Antoine Stier, Quentin Schull, Pierre Bize, Emilie Lefol, Mark Haussmann, Damien Roussel, Jean-Patrice Robin, Vincent A. Viblanc

**Affiliations:** 1 Department of Biology, University of Turku, Turku, Finland; 2 Institute of Biodiversity, Animal Health and Comparative Medicine, University of Glasgow, Glasgow, UK; 3 Université d’Angers, Angers, France; 4 Université de Strasbourg, CNRS, IPHC UMR 7178, 67000 Strasbourg, France; 5 School of Biological Sciences, University of Aberdeen, Aberdeen, UK; 6 Département de Biologie, Faculté des Sciences, Université de Sherbrooke, Sherbrooke, Canada; 7 Department of Biology, Bucknell University, Lewisburg, USA; 8 Laboratoire d’Ecologie des Hydrosystèmes Naturels et Anthropisés, CNRS UMR 5023, Université de Lyon, Lyon, France

**Keywords:** Ecophysiology, Animal physiology

## Abstract

Exposure to unpredictable environmental stressors could influence animal health and fitness by inducing oxidative stress, potentially through downstream effects of glucocorticoid stress hormones (*e.g*. corticosterone) on mitochondrial function. Yet, it remains unclear whether species that have evolved in stochastic and challenging environments may present adaptations to alleviate the effects of stress exposure on oxidative stress. We tested this hypothesis in wild king penguins by investigating mitochondrial and oxidative stress responses to acute restraint-stress, and their relationships with baseline (potentially mirroring exposure to chronic stress) and stress-induced increase in corticosterone levels. Acute restraint-stress did not significantly influence mitochondrial function. However, acute restraint-stress led to a significant increase in endogenous antioxidant defences, while oxidative damage levels were mostly not affected or even decreased. High baseline corticosterone levels were associated with an up-regulation of the glutathione antioxidant system and a decrease in mitochondrial efficiency. Both processes might contribute to prevent oxidative damage, potentially explaining the negative relationship observed between baseline corticosterone and plasma oxidative damage to proteins. While stress exposure can represent an oxidative challenge for animals, protective mechanisms like up-regulating antioxidant defences and decreasing mitochondrial efficiency seem to occur in king penguins, allowing them to cope with their stochastic and challenging environment.

## Introduction

Vertebrates respond to stressful and unpredictable environmental stimuli (*e.g*. food shortage, predation, adverse weather conditions, etc.) by activating a set of physiological and behavioural responses defined as a whole as the ‘stress response’^1^. One key component of the stress response is the activation of the hypothalamic-pituitary-adrenal (HPA) axis, ultimately leading to the release of glucocorticoid (GC) hormones in the blood stream. GCs are a group of steroid hormones, central to the regulation of energy balance in vertebrates^1,2^. At baseline levels they are involved in the regulation of energy balance associated with both the predictable demands of life-history cycles such as seasonal and diurnal variations^2,3^, and the prolonged exposure to environmental stressors^4,5^. In contrast, acute increases in GC allow animals to maintain homeostasis when faced with unpredictable environmental events (*e.g*. predation, detrimental weather^1,6^). A key feature of GCs is their role in redirecting energy allocation from non-essential to essential functions, by regulating energy intake, processing and expenditure^2^.

Elevated baseline GC levels may reflect adaptive responses (*e.g*. stress responsiveness) of individual to cope with specific life-history stages or challenges, but may also reflect situations of chronic stress when organisms are continuously exposed to stressful stimuli that may ultimately affect their fitness^7^. However, the relationship between baseline GC levels and fitness is not consistent^8^, and little is known about the physiological pathways relating GCs to individual fitness. Recently, oxidative stress has been suggested as one important down-stream consequence of chronically elevated baseline GC levels, with potential consequences on fitness^9–11^. Oxidative stress is a complex, multi-faceted state that arises in organisms as a consequence of the imbalance between the production of pro-oxidant molecules and antioxidant defences^12,13^. Reactive oxygen species (ROS) are one main type of pro-oxidants, and partly arise as by-products of cellular respiration in mitochondria^14^. Although ROS have important roles in cell signalling, they act as a double-edged sword damaging macromolecules, cell components and structures when produced in excess^12–14^. In the past decade several studies have highlighted direct links between chronic stress exposure and/or chronically elevated baseline GC levels and oxidative stress^9,15–17^. There is also growing evidence that acute stress exposure could cause oxidative stress. Accordingly, it has been shown that acute restraint-stress (≤1 hour) could increase ROS production^18,19^, oxidative damage to lipids and DNA^20–22^, while having mixed effects on antioxidant defences^18–24^. Most of those studies were however conducted in laboratory models adapted to constant and unchallenging environments (*i.e*. food ad-libitum, no predation risk, controlled microclimate, etc.). Hence, we still know little on the effects of stress and the GC response on oxidative stress in the wild. In particular, whether species that evolved in stochastic and challenging environments may present adaptations to cope with stress-induced oxidative stress is unknown.

Since mitochondria are the site of aerobic respiration and one major sources of ROS, they have been suggested to play a key role in the stress response^25,26^. This idea has however received limited attention so far, even though mitochondrial dysfunction may constitute a central pathway linking stress exposure to impaired organismal maintenance^27^. The presence of GC receptors in mitochondria suggests that they may play a role in the stress response^28^. GCs are known to affect mitochondrial gene expression, mitochondrial biogenesis, and mitochondrial fission/fusion dynamics, influencing both ATP and ROS production^25,26^. However, the way GCs affect mitochondrial function could differ depending on GC dose and duration of exposure (*e.g*. differences between acute and chronic GCs exposure^25,29^). For instance, exposure to moderate or high GC levels increases mitochondrial oxidative capacity in the short-term, but prolonged exposure to high GC levels decreases mitochondrial oxidative capacity^29^. Additionally, GCs have been shown to increase potentially both mitochondrial and non-mitochondrial sources of ROS production^30^. While we ignore the fine time scale (*i.e*. seconds, minutes, hours) at which GCs could affect mitochondrial function *in vivo*, other hormones such as thyroid hormones have been shown to have very rapid effects (<1 hour) on mitochondrial respiration^31^. Therefore, rapid changes in mitochondrial function could be part of the acute stress response. Our current knowledge is mostly based on studies using cultured cells, and we lack information on the links between mitochondrial function and GC stress responses at the organism level, particularly in species evolving in their natural environment. The sensitivity of mitochondria to GCs could vary between strains of the same captive species^32^. Thus, it is possible that the relationships between GCs, mitochondrial function, and oxidative stress (see above) could differ between species, life history stages, and environmental conditions.

We tested this hypothesis in freely-breeding king penguins (*Aptenodytes patagonicus*) by investigating mitochondrial and oxidative stress responses to acute restraint-stress, and their relationships with baseline (potentially mirroring exposure to chronic stress) and stress-induced increase in corticosterone levels. King penguins are an interesting model to investigate the oxidative and mitochondrial responses to stress exposure. These sub-Antarctic seabirds breed on-land while being exposed to numerous environmental stressors (predation, parasites, detrimental weather, aggressive social environment^33–35^). They have evolved both behavioural and physiological adaptations to tolerate periods of long-term fasting^36^ (including mitochondrial adaptations^37^ and adaptations to oxidative stress^38^). Finally, our studies show that baseline corticosterone (CORT) levels in this species are positively associated with chronic stressors such as high intra-specific density in an aggressive social environment^39^ and ectoparasites prevalence (Bize et *al*., unpublished).

Here, we subjected 24 free-living incubating king penguins to an acute restraint-stress for 30 minutes. We collected blood samples both before (baseline <4 min post-capture^40^) and at the end of the 30 minutes of restraint-stress. We used these samples to measure baseline and stress-induced plasma total CORT levels and oxidative stress markers measured both in plasma and red blood cells (RBCs). Birds also have functional mitochondria in their RBCs^41,42^, thus allowing us to assess short-term changes in mitochondrial function by repeated blood sampling^43^. First, we investigated the impact of acute restraint-stress on oxidative stress levels and mitochondrial function. Second, we tested if between-individual differences in CORT response (*i.e*. ΔCORT: T30 – baseline) could predict between-individual differences in oxidative stress and mitochondrial responses to restraint-stress. Indeed, if the acute rise in GCs is the mechanism leading to changes in mitochondrial function and oxidative stress during acute stress, we would expect that birds having a higher CORT response would also show more pronounced changes in oxidative stress levels and mitochondrial function. Finally, we assessed the relationships between baseline CORT levels (potentially mirroring exposure to chronic stress) and both baseline oxidative stress levels and mitochondrial function.

Given the numerous environmental challenges faced by king penguins while breeding on land and foraging at sea, we hypothesized that this bird species should have evolved specific adaptations alleviating the effects of stress exposure and/or high GC levels on oxidative stress.

## Material and Methods

### Study species and sampling procedures

This study took place on Possession Island in the Crozet Archipelago (46°25′S; 51°52′E). Data were collected during the 2013–2014 breeding season in the king penguin colony of “La Grande Manchotière” (*ca*. 24,000 breeding pairs). This study has been approved by the ethical committee ‘Comité d’éthique de la Fédération de Recherche en Biologie de Toulouse’ (C2EA-01 FRBT), project N°119-2013, and all experiments were performed in accordance with relevant guidelines and regulations. During the breeding season, male and female king penguin alternate between periods on-land caring for the egg or chick, and periods foraging at sea^44^. After a courtship period of *ca*. 15-days on land, the female lays a single egg and the male takes care of the first incubation shift while the female leaves to forage at sea. She returns to relieve her partner *ca*. 15-days later. Alternated guards continue throughout incubation (53 days) and early chick brooding. In this study, we caught females (N = 12) and males (N = 12) from independent breeding pairs at the beginning of their incubation shift. Females were caught at the start of their first incubation shift, and males were caught at the start of their second incubation shift to ensure both sexes had experienced similar fasting durations (birds were all caught 3 days after their return from sea). This was particularly important given the known effects of long-term fasting on both baseline CORT and oxidative stress levels in this species^38,45^.

Birds caught during incubation were rapidly blood-sampled after capture (<4 min; mean ± SE = 3′22″ ± 11″) after placing the egg in an insulated box and replacing it by a dummy to avoid accidental breakage during handling. Blood (*ca*. 1 mL) was taken from the flipper vein using a heparinized syringe. In the king penguin, no detectable increase in baseline GC levels occurs within the first 5 minutes of capture^40^. Thus, corticosterone levels measured in this first blood sample represent bird’s baseline levels under natural conditions. Birds were manually restrained by the same experimenter (EL) and a second blood sample (*ca*. 1 mL) was taken *ca*. 30 min post-capture (mean ± SE = 31′22″ ± 14″). All birds continued to incubate at the end of the restraint-stress period, and our manipulations never resulted in breeding failure. All blood samples were kept on crushed ice until centrifugation (30–60 min later) at 3000 *g* for 10 min to separate plasma from RBCs. The plasma fraction was then removed and plasma aliquots were stored at −20 °C until the end of the day, before being transferred to −80 °C until laboratory analyses. Two aliquots of 100 μL of pelleted RBCs were transferred into a new tube containing 1 mL of phosphate buffer saline (PBS) at 4 °C. After gentle homogenisation, RBCs were washed a first time by centrifuging the samples at 600 *g* for 5 min to pellet the cells and discard the supernatant. RBCs were then re-suspended in 1 mL of ice-cold PBS, and one aliquot was frozen at −80 °C for later analysis of oxidative stress markers, while the second was stored at 4 °C until being used for mitochondrial measurements (see below). At this time, samples were washed a second time as described above and re-suspended in 1 mL of respiratory buffer MiR05 (0.5 mM EGTA, 3 mM MgCl_2_, 60 mM K-lactobionate, 20 mM taurine, 10 mM KH_2_PO_4_, 20 mM Hepes, 110 mM sucrose, free fatty acid bovine serum albumin (1 g/L), pH 7.1).

### Corticosterone (CORT) measurement

Plasma total CORT levels were determined by immunoassay according to guidelines provided by the manufacturer (Corticosterone EIA Kit, Enzo Life Sciences, USA) and as previously used in this species^46,47^. Intra-individual coefficient of variation based on duplicates was 9.86 ± 2.98%.

### Oxidative stress measurements

We evaluated a total of 14 markers of oxidative status (*i.e*. both oxidative damage and antioxidant defences), 6 in plasma and 8 in RBCs (see Table 1 for a brief overview). Although blood samples might not necessarily always reflect the whole-body response in terms of oxidative stress, we combined two sample types providing different information (*i.e*. plasma being an extra-cellular compartment integrating responses from different organs and RBC being a specific cell type but possessing the full cellular machinery) to make the most of this non-invasive and non-lethal sampling. Technical precision of measurements (CVs in %) was evaluated based on 6 samples being analysed in duplicate (3 baseline and 3 T30 samples).

**Table 1:** Summary of physiological parameters measured in this study and their biological meaning.

Physiological parameters		Biological meaning
Glucocorticoids	*Baseline CORT*	Baseline corticosterone levels
	*CORT T30*	Corticosterone absolute stress response to 30 min of standardized handling
	*ΔCORT*	Corticosterone relative stress response to 30 min of handling (T30 minus baseline)
Oxidative damage	*Plasma ROMs*	Reactive Oxygen Metabolites in the plasma, a marker of early oxidative damage
	*Plasma protein carbonyl*	Oxidative damage to proteins in the plasma (*i.e*. extra-cellular)
	*Plasma 8-OHdG*	Circulating levels of 8-OHdG, a product of oxidative damage to DNA reflecting whole-body oxidative stress and being influenced by rates of both damage and repair
	*RBC protein carbonyl*	Oxidative damage to proteins in the red blood cells (*i.e*. intra-cellular)
	*RBC 8-OHdG*	Oxidative damage to DNA being integrated into cellular DNA of red blood cells
	*RBC GSSG*	Glutathione in red blood cells that has been oxidized by reactive oxygen species
	*RBC GSSG/tGSH*	Proportion of glutathione in red blood cells being oxidized relative to the overall quantity of glutathione
Antioxidant defences	*RBC tGSH*	Red blood cell total (reduced + oxidized) content of the endogenous antioxidant glutathione
	*RBC GPx*	Activity of the endogenous antioxidant enzyme glutathione peroxidase in red blood cell
	*RBC SOD*	Activity of the endogenous antioxidant enzyme superoxide dismutase in red blood cell
	*RBC OXY*	Total content of non-enzymatic antioxidants in red blood cells
	*Plasma GPx*	Activity of the endogenous antioxidant enzyme glutathione peroxidase in plasma
	*Plasma SOD*	Activity of the endogenous antioxidant enzyme superoxide dismutase in plasma
	*Plasma OXY*	Total content of non-enzymatic antioxidants in the plasma (mostly acquired through the diet)
Mitochondrial respiration rates of intact RBCs	*ROUTINE*	Mitochondrial respiration of intact red blood cells with their endogenous substrates and ADP
	*OXPHOS*	Mitochondrial respiration linked to ATP synthesis
	*LEAK*	Mitochondrial respiration linked to mitochondrial proton leak
	*ETS*	Maximal mitochondrial respiration of intact red blood cells with their endogenous substrates
Mitochondrial Flux Control Ratios of intact RBCs	*FCR*_*L/R*_	Proportion of mitochondrial ROUTINE respiration being linked to proton leak, indicating mitochondrial efficiency under endogenous cellular conditions
	*FCR*_*L/ETS*_	Proportion of mitochondrial maximal respiration being linked to proton leak, indicating mitochondrial efficiency under stimulated cellular conditions
	*FCR*_*R/ETS*_	Proportion of mitochondrial maximal respiration being used under endogenous cellular conditions, indicating the activation state of the mitochondria

#### DNA oxidative damage

8-OHdG is one of the predominant forms of free radical-induced oxidative lesions on DNA. At the cellular level, damaged guanine (8-OHdG) can be excised from genomic DNA by specific repair enzymes, and enters the circulation before being eliminated through urine. Consequently, plasma and urinary levels of this marker have the potential to reflect whole-body oxidative stress status, and are both influenced by the level of damage and by the rate of repair of such damage^12^.

The circulating concentration of 8-OHdG was quantified using a competitive immunoassay (plasma diluted 1:5; DNA damage ELISA Kit, Enzo® Life Sciences, USA) as previously described in king penguin^39,46^. Plasma DNA damage is expressed as ng of 8-OHdG/mL, and intra-individual coefficient of variation based on duplicates was 12.44 ± 2.05%.

The 8-OHdG incorporated in RBC DNA was quantified using a competitive immunoassay (300 ng DNA, EpiQuick 8-OHdG DNA Damage Quantification Direct Kit Colorimetric, Epigentek, USA). Briefly, DNA was extracted from 40 μL of RBC-PBS solution using a commercial spin-column method (NucleoSpin Blood QuickPure, Macherey-Nagel, Germany) and quantified spectrophotometrically using a NanoDrop^TM^ (ThermoFisher Scientific^TM^). Then, 300 ng of purified DNA was used in the ELISA assay that was conducted following manufacturer recommendations. RBC DNA damage is expressed as pg of 8-OHdG/μg of DNA, and intra-individual coefficient of variation based on duplicates was 6.20 ± 1.19%.

#### Reactive oxygen metabolites (ROMs)

The concentration of reactive oxygen metabolites (ROMs) in the plasma was measured using the d-ROM tests (5 µL of plasma, Diacron International, Italy) following the manufacturer instructions and as previously used in king penguin^39,46^. The d-ROM test measures mostly hydroperoxides (ROOH) as a marker of potential oxidative damage and has been validated and extensively used in the past decade in birds^48^. ROMs concentration is expressed as mg of H_2_O_2_ equivalent/dL and intra-individual variation based on duplicates was 7.45 ± 1.03%.

#### Protein carbonyl content

Carbonyl groups are introduced into the proteins by reactions with free radicals or lipid peroxidation products, and damage produced by protein carbonylation is mostly irreversible^12^. The carbonyl content of plasma and RBC samples (diluted to 1 mg protein.mL^−1^) was quantified using a previously published protocol^49^. First, nucleic acids were removed by precipitation with streptomycin sulphonate (15 min at room temperature) and centrifugation at 12,000 *g* for 10 min. Protein carbonyls were then derivatized to 2,4-dinitrophenylhydrazone by reaction with 2,4-dinitrophenylhydrazine (DNPH) for 1 h at room temperature. The pellet was precipitated with cold trichloroacetic acid at 20% and then washed three times with a 1:1 solution of cold ethanol:ethyl acetate. The pellet was finally re-suspended in 350 μL of guanidine hydrochloride 6M. The absorbance of the samples was read at 370 nm and the mean absorbance of control tubes (incubated with 0.1 M HCl instead of DNPH) was then subtracted. We used the extinction coefficient of DNPH (0.022 μmol.L^−1^.cm^−1^) to calculate protein carbonyl content, which was expressed as nmol.mg^−1^ of protein. Intra-individual variation based on duplicates was 14.58 ± 2.27%.

#### Superoxide dismutase (SOD) and glutathione peroxidase (GPx) antioxidant activities

SOD is involved in the first step of the antioxidant enzymatic cascade catalysing the dismutation of superoxide radical into oxygen and hydrogen peroxide. The enzymatic activity of SOD in plasma (diluted 1:6) and RBC lysate (diluted 1:500) was measured with the SOD activity kit (Enzo® Life Sciences, USA) following manufacturer instructions. This test quantifies *in vitro* the kinetics of inhibition in superoxide formation resulting from SOD antioxidant activity. SOD activity is expressed as U (units of enzymatic activity).mL^−1^. Intra-individual coefficient of variation based on duplicates was 12.00 ± 1.96%.

Glutathione is used as a reductant by the GPx enzyme to scavenge deleterious hydrogen peroxide. The enzymatic activity of GPx in plasma (diluted 1:10) and RBC lysate (diluted 1:60) was measured with the RANSEL kit (Randox Laboratories, Crumlin, UK) following manufacturer instructions. GPx activity is expressed as U.L^−1^. Intra-individual coefficient of variation based on duplicates was 9.09 ± 1.40%.

#### Total and oxidized glutathione

Total glutathione (tGSH) content and oxidized glutathione (GSSG) content of RBC lysate (diluted 1:480) were determined using DetectX® Glutathione fluorescent detection kit (Arbor Assays, USA), following manufacturer instructions. Glutathione plays a key role in many biological processes including the protection of cells against oxidation. We evaluated tGSH content as an indicator of antioxidant protection, the amount of GSSG, and the ratio GSSG/tGSH (which represent the proportion of oxidized glutathione) as indicators of the oxidative challenge (*i.e*. the pro-oxidant power buffered by the glutathione system). Values are respectively expressed as mmol.L^−1^, and as a ratio of GSSG/tGSH (0 meaning that all glutathione is reduced GSH, and 1 meaning that all glutathione is oxidized). Intra-individual coefficient of variation based on duplicates was 1.41 ± 0.41%.

#### Non-enzymatic (NE) antioxidant capacity OXY

We evaluated the NE antioxidant capacity of plasma (diluted 1:100) and red blood cell lysate (diluted 1:2500) using the OXY-adsorbent test (Diacron International, Italy) following manufacturer instructions. The OXY adsorbent test quantifies the ability of NE antioxidant compounds to buffer a massive oxidation through hypochlorous acid (HClO). This assay measures a variety of NE antioxidants, including vitamins, carotenoids, flavonoids and thiols. NE antioxidant capacity is expressed as μM of HClO neutralized.L^−1^ for the plasma and as mM of HClO neutralized.L^−1^ for RBCs. Intra-individual coefficient of variation based on duplicates was 5.86 ± 0.98%.

### Mitochondrial measurements in RBCs

We followed the protocol we recently established for king penguins^43^ to measure mitochondrial function in fresh intact RBCs using a high-resolution respirometry system O2k (Oroboros Instrument, Innsbruck, Austria) at the temperature of 38 °C. We applied a serial addition of chemicals to our RBC suspension in order to get a comprehensive assessment of mitochondrial function. We used 2.5 µM of oligomycin to inhibit ATP synthesis, then 1 µM of the mitochondrial uncoupler FCCP to stimulate respiration and finally 5 µM of Antimycin A to fully inhibit mitochondrial respiration, as previously described^43^. We computed four parameters of mitochondrial respiration (*ROUTINE*, *OXPHOS*, *LEAK* and *ETS*) that were normalized by the fresh mass of RBCs, and three different flux control ratios (FCRs) to better characterize mitochondrial function of RBCs (see Table 1 and^43^ for details). Mitochondrial respiration in intact cells is likely to be substrate and ADP limited, thereby not reflecting maximal mitochondrial capacity. While our results in other bird species comparing intact and permeabilized RBCs support this idea, they also suggest that mitochondrial parameters measured in both conditions are highly correlated (Stier & Bize, pers. obs.). A summary of all physiological markers is presented in Table 1 and original data is available at 10.6084/m9.figshare.7042049. All chemicals were purchased from Sigma-Aldrich (France) unless specified otherwise.

### Statistics

All statistical analyses were conducted using SPSS 20.0. We investigated the effects of the acute restraint stress on corticosterone, mitochondrial parameters and oxidative stress markers using repeated-measures Generalized Estimating Equations (GEE) with a Gaussian distribution, with bird ID as the individual factor, sampling time (*i.e*. baseline *vs*. acute stress) as the within-individual repeated effect, and sex as a fixed factor. We initially included the interaction between sampling time and sex, but removed it from the final models since the interaction was never significant (all p > 0.07). Standardized effect sizes *d* and their 95% confidence interval for acute stress response were calculated following equations (4) and (18)^50^.

We investigated relationships between the magnitude of the relative CORT response (Δ CORT) and the magnitude of both oxidative stress and mitochondrial responses to acute restraint-stress (Δ oxidative stress and Δ mitochondria) in separate analyses. We used General Linear Models (GLMs) with sex and Δ CORT as explanatory variables. Finally, we investigated relationships between baseline CORT and respectively baseline mitochondrial parameters and oxidative stress markers using GLMs with sex and baseline CORT as explanatory variables. In both cases we calculated standardized effect sizes *Zr* and their 95% confidence interval using equations (11), (19) and (20)^50^.

While measuring a broad range of physiological markers (*i.e*. 21 in our case, see Table 1) brings detailed and valuable biological information, it comes at the cost of increasing the number of statistical tests to perform, therefore increasing the likelihood of type I statistical errors (*i.e*. false positives). Correcting for multiple testing comes at the cost of decreasing considerably statistical power^51^, which in the case of experimental studies on wild animals like king penguins could not be overcome by increasing substantially sample size for ethical and practical constraints. We therefore chose to present results mostly as effect size reflecting the magnitude of the biological effects we observe (Nakagawa *et al*. 2004), but still provide the reader with information on p-values after false discovery rate (FDR) correction. We used the two-stage sharpened method for FDR^52^.

Sample sizes vary slightly between markers due to 2 missing plasma samples and a few failed laboratory assays (see full dataset available as ESM). Means are given ± SE and p-values ≤ 0.05 were considered as significant.

## Results

### Corticosterone response to acute restraint-stress

Plasma total CORT was on average 12.7 times higher in response to our standardized restraint-stress protocol than at baseline levels (Wald χ^2^ = 25.05, p < 0.001, Fig. S1). We found no significant effect of sex (Wald χ^2^ = 2.54, p = 0.11) on the CORT response.

### Oxidative stress response to acute restraint-stress

**Figure 1:**
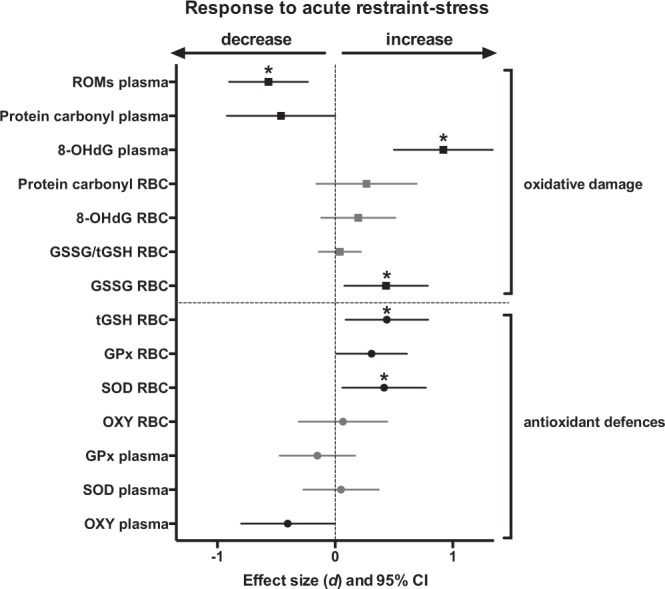
Effects of acute restraint-stress on oxidative stress parameters. Standardized effect sizes *d* and their 95% confidence intervals are shown. Statistically significant effects (95% CI not overlapping 0) are presented in black, while non-significant ones (95% CI overlapping 0) are presented in light grey. Details on statistical tests are provided in Table S1. Parameters remaining significant after false discovery rate (FDR) correction are marked with a *.

Eight out of 14 oxidative stress markers were significantly affected by our standardized restraint-stress protocol (Figs 1, S2, S3 and Table S1).

#### Oxidative damage

In the plasma, two markers of oxidative damage (ROMs and protein carbonyl) decreased in response to acute restraint-stress, while 8-OHdG increased (Figs 1 and S2). In RBCs, our two markers of oxidative damage were not significantly influenced by acute restraint-stress, and while oxidized glutathione (GSSG) content increased in response to acute stress, this was not the case for the proportion of glutathione oxidized (GSSG/tGSH). Females had higher ROMs values (females = 6.81 ± 0.62, males = 3.17 ± 0.38 mg of H_2_O_2_ equivalent/dL) but lower RBC 8-OHdG levels (females = 59.41 ± 3.31, males = 73.49 ± 5.32 pg of 8-OHdG/μg of DNA) than males; other parameters did not significantly differ between sexes (Table S1). The effect of acute restraint-stress on plasma protein carbonyl was not significant anymore after FDR correction.

#### Antioxidant defences

Plasma NE antioxidants decreased in response to acute restraint-stress, while plasma SOD and GPx were not significantly impacted (Figs 1 and S3). In RBCs, we found an increase in 3 markers of endogenous antioxidant defences (total glutathione, SOD and GPx) in response to acute restraint-stress, while NE antioxidants were not significantly affected (Fig. 1, Table S1). Markers of antioxidant defences were overall not significantly different between males and females, except for plasma SOD with males having higher levels than females (males = 16.31 ± 0.68, females = 9.58 ± 0.49 U.mL^−1^). The effects of acute restraint-stress on plasma NE antioxidants and RBC GPx were not significant anymore after FDR correction.

**Figure 2:**
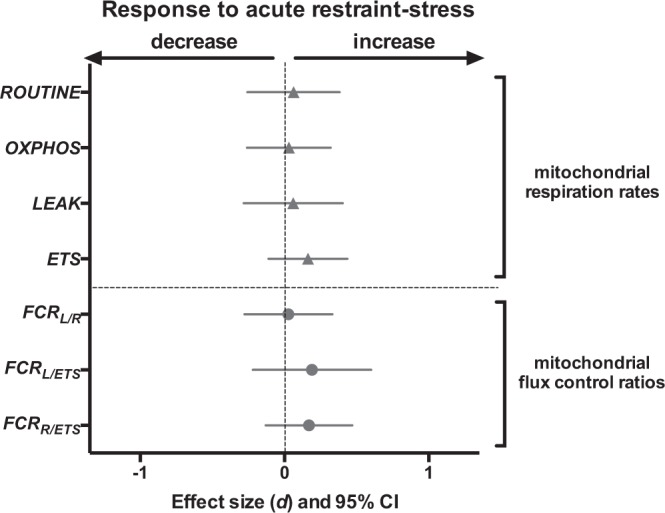
Effects of acute restraint-stress on mitochondrial parameters. Standardized effect size *d* and their 95% confidence interval are shown. Non-significant effects are presented in light grey. Details on statistical tests are provided in Table S1.

### Mitochondrial response to acute restraint-stress

We found no significant effect of our standardized restraint-stress protocol (all p > 0.22, Figs 2 and S3) or sex (all p > 0.14) on mitochondrial parameters (Table S1).

### Relationships between acute CORT response and acute changes in oxidative stress and mitochondrial function in response to restraint-stress

**Figure 3:**
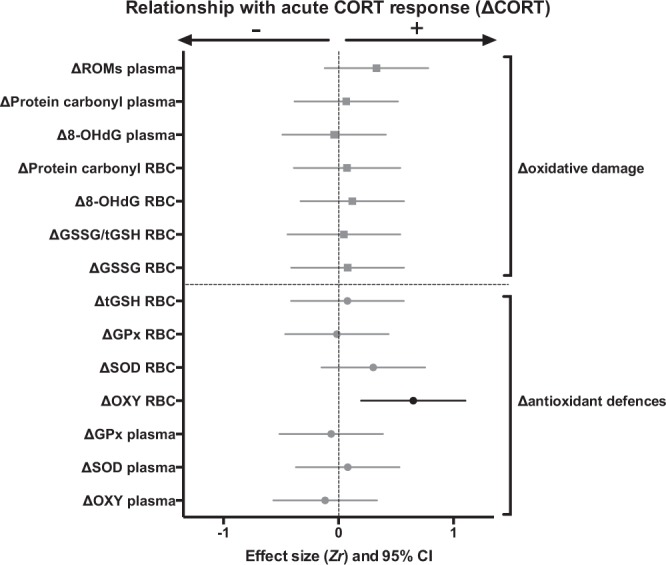
Relationships between corticosterone (CORT) and oxidative stress responses to an acute restraint-stress. Standardized effect size *Zr* and their 95% confidence interval are shown. Statistically significant effects are presented in black, and non-significant ones in light grey. Details on statistical tests are provided in Table S2. Parameters remaining significant after false discovery rate (FDR) correction are marked with a *.

The magnitude of the CORT response (ΔCORT) was overall not predictive of the magnitude of the changes in oxidative stress markers in response to acute restraint stress (Δoxidative stress), except for RBC NE antioxidants (Fig. 3 and Table S2). Birds having a marked CORT response also had a positive change in RBC NE antioxidants in response to acute restraint-stress, although this effect became non-significant after FDR correction.

**Figure 4:**
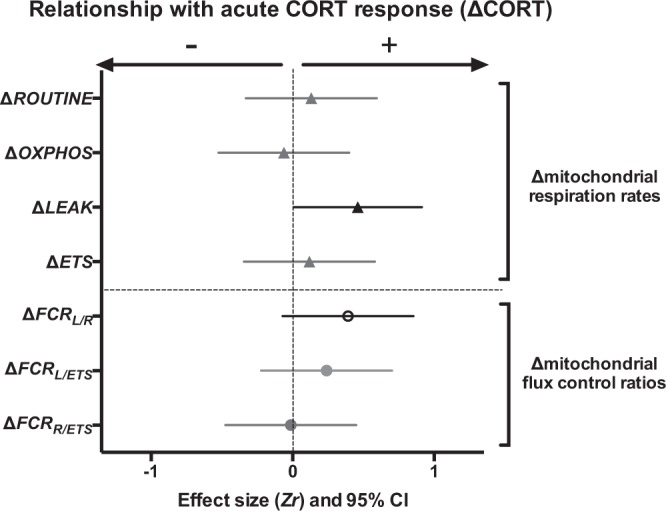
Relationships between corticosterone (CORT) and mitochondrial stress responses to an acute restraint-stress. Standardized effect size *Zr* and their 95% confidence interval are shown. Statistically significant effects are presented in black, marginally-significant ones (p ≤ 0.10) in dark grey with open symbol, and non-significant ones in light grey. Details on statistical tests are provided in Table S2. Parameters remaining significant after false discovery rate (FDR) correction are marked with a *.

The magnitude of the CORT response (ΔCORT) was overall not predictive of the magnitude of the changes in mitochondrial function in response to acute restraint stress (Δmitochondria), except for *LEAK* respiration (Fig. 4 and Table S2). Birds having a marked CORT response tended to have a positive change in *LEAK* respiration in response to acute restraint-stress, although this effect became non-significant after FDR correction.

### Relationships between baseline CORT and baseline oxidative stress levels

**Figure 5:**
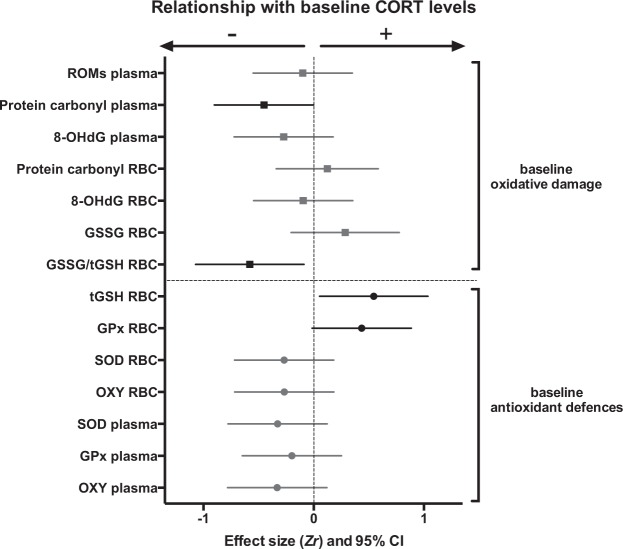
Relationships between baseline levels of corticosterone (CORT) and oxidative stress markers. Standardized effect size *Zr* and their 95% confidence interval are shown. Statistically significant effects are presented in black, marginally-significant ones (p ≤ 0.10) in dark grey with open symbol, and non-significant ones in light grey. Details on statistical tests are provided in Table S3. Parameters remaining significant after false discovery rate (FDR) correction are marked with a *.

We found a positive relationship between baseline CORT and RBC total glutathione content, and a marginally significant positive relationship between baseline CORT and RBC GPx antioxidant activity (Fig. 5, Table S3). Baseline CORT was also negatively correlated with two markers of oxidative damage, namely RBC proportion of oxidized glutathione and plasma protein carbonyl. These effects did not remain significant after FDR corrections. All other relationships between baseline CORT and baseline oxidative stress parameters were non-significant (Fig. 5, Table S3).

### Relationship between baseline CORT and baseline mitochondrial parameters

**Figure 6:**
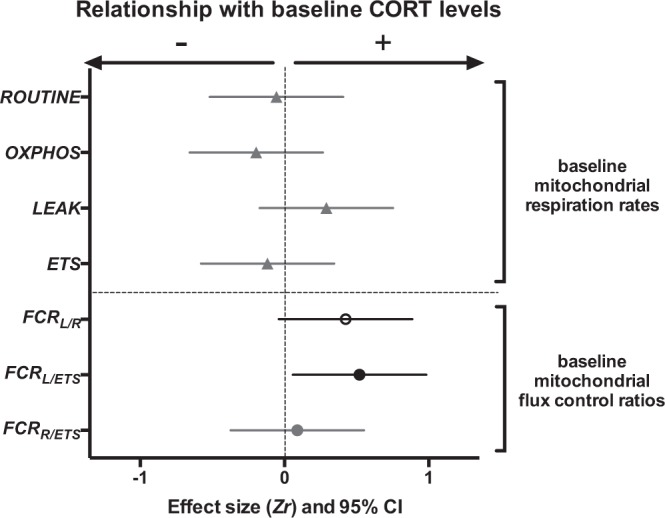
Relationships between baseline levels of corticosterone (CORT) and mitochondrial parameters. Standardized effect size *Zr* and their 95% confidence interval are shown. Statistically significant effects are presented in black, marginally-significant ones (p ≤ 0.10) in dark grey with open symbol, and non-significant ones in light grey. Details on statistical tests are provided in Table S3. Parameters remaining significant after false discovery rate (FDR) correction are marked with a *.

Baseline CORT was not significantly related to mitochondrial respiration rates (Fig. 6, Table S3). However, we found a marginally significant positive relationship between baseline CORT and *FCR*_*L/R*_ and a significant positive relationship between baseline CORT and *FCR*_*L/ETS*_, showing that individuals with higher baseline CORT had less efficient mitochondria (Fig. 6, Table S3). However, these effects did not remain significant after FDR corrections.

## Discussion

We tested the hypothesis that king penguin, a colonial seabird species subject to challenging environmental conditions and stressful stimuli while breeding on-land^33,35,53^, could be naturally equipped to prevent stress-induced or GC-induced oxidative stress. We investigated mitochondrial and oxidative stress responses to acute restraint-stress, and their relationships with baseline (potentially mirroring exposure to chronic stress) and stress-induced increase in corticosterone levels. In an attempt to achieve an integrative perspective on individual mitochondrial and oxidative responses to stress, we measured both baseline and stress-induced GC levels and investigated their relationship with an exhaustive number of physiological markers (14 oxidative status markers and 7 markers of mitochondrial functions). It follows that because of limited number of sampled birds (N = 24) – one of the constraints of working on wild protected species – some of the effects we document became non-significant after FDR correction. Yet, what is important to note is that FDR changes the 95% confidence intervals around estimates, not the estimates themselves. Thus, we encourage readers to think about the meaning of effect sizes relative to one another, rather than focusing solely on *p*-values^50^. We nonetheless point out to effects that were no longer significant after FDR in the following discussion.

Our results show an increase in endogenous antioxidant defences both in response to acute restraint-stress and in relationship with increased baseline GC levels, whereas oxidative damage levels were mostly reduced or not affected. We also found a reduction in a proxy of mitochondrial efficiency to produce ATP (*i.e*. higher proportion of respiration being linked to mitochondrial proton leak) both in relationship to the magnitude of the acute CORT response and with increasing levels of baseline CORT. Because lower mitochondrial efficiency is suggested to reduce ROS production^54^, this suggests that an increase in acute CORT response or in baseline CORT may be associated with a reduction in mitochondrial ROS production in this species. Mitochondrial function was however not affected by acute restraint-stress. King penguins seem therefore well equipped to buffer potential oxidative damage arising from acute stress or high GC exposure, which contrasts to what has been found in many other animals to date^9,11,18,20–22^. Most of our knowledge so far on the mechanisms linking acute/chronic stress or high GC exposure to oxidative stress comes from laboratory animals adapted to unchallenging controlled environments. Therefore, our study illustrates the importance of studying non-traditional model species in their natural habitat to provide a comprehensive overview of the influence of stress exposure on both oxidative stress and mitochondrial function, and to gain knowledge on how some species may thrive in seemingly stressful environments.

### Acute stress: relationships between GCs, mitochondrial function and oxidative stress

As expected, adult penguins experimentally restrained for a 30-minute-period experienced a substantial increase in total CORT levels. However, this acute restraint had no significant impact on mitochondrial function in RBCs at the population level, on average. Yet, birds with the higher CORT response (ΔCORT) tended to be the ones exhibiting an increase in mitochondrial proton leak in response to acute stress (Δ*LEAK*), which could contribute in counter-balancing the potential pro-oxidant effects of high CORT during acute stress (see below for details on mitochondrial uncoupling and ROS mitigation^54,55^). Unfortunately, we were not able to measure mitochondrial ROS production *per se*, and information in other tissues than RBCs will also be needed to better understand the potential role of mitochondria in the acute stress responses. Tissues can highly differ in their sensitivity to GCs^56^, so it is possible that acute increases in GCs may have more pronounced effects on mitochondria from tissues being more directly involved in the “fight or flight” response, such as skeletal muscles. Despite the absence of modifications in mitochondrial parameters after 30 minutes of restraint stress at the average population level, it is possible that effects could become evident after a more prolonged restraint stress, or even during/following the recovery from this acute stress.

Oxidative damage levels changed markedly in response to acute restraint-stress (4 markers out of 7). Surprisingly, two markers of oxidative damage decreased in response to acute stress. These findings contrast with the idea that acute stress may increase oxidative damage^20–22^, and might be explained by the concurrent increase in several antioxidant mechanisms (3 markers out of 7; see below). Nevertheless, we found an increase in plasma 8-OHdG, but not in RBC 8-OHdG, in response to acute stress. We have to keep in mind that plasma 8-OHdG integrates whole-body DNA damage, but is also influenced by repair levels. Therefore, two non-mutually exclusive hypotheses could explain these results. First, elevated plasma 8-OHdG levels may indicate increased repair of DNA damage at the cellular level to keep 8-OHdG integrated within genomic DNA at a constant level, explaining the lack of increase in RBC 8-OHdG. Second, DNA damage levels in response to acute stress may differ between RBCs and other tissues. Hence, an increase in plasma 8-OHdG levels may reflect increased oxidative stress in other tissues than RBCs, potentially in some tissues being more sensitive to acute stress (*e.g*. skeletal muscle). Finally, the elevated levels of oxidized glutathione we found in response to acute stress could be indicative of an increased amount of ROS being quenched by the glutathione system. Whether these ROS could originate from the mitochondria or from other sources (*e.g*. autoxidation of haemoglobin or activity of enzymes such as xanthine oxidase) remains nonetheless unknown, calling for direct measurement of both mitochondrial and non-mitochondrial ROS production during acute stress in the future.

Antioxidant defences were also affected by acute stress (4 markers out of 7). The only marker that decreased in response to acute stress was plasma non-enzymatic antioxidant (sometimes referred to as TAC, total antioxidant capacity), which is in accordance with previous studies in birds^19–21,24^. This antioxidant marker reflects mostly antioxidant compounds acquired through the diet^57^, and the observed decrease in plasma TAC probably reflected their consumption to buffer increase in ROS production resulting from acute stress. In contrast, several RBC antioxidant systems (glutathione, GPx and SOD) increased, suggesting an activation of cellular endogenous antioxidant defences in response to acute stress. This activation of endogenous antioxidants might have been greater than the potential increase in ROS, thereby explaining why both plasma ROMs and protein carbonyl levels decreased in response to acute restraint-stress.

We found no significant relationship between the intensity of the acute CORT response and the magnitude of the oxidative stress response (Fig. 3) except for RBC NE antioxidants, suggesting that these two processes are potentially independently regulated. Yet, experiments inducing an acute increase in exogenous GCs (without inducing restraint-stress) and measuring oxidative stress levels in response to this challenge are needed to exclude a direct role of GCs. Beside GCs, other hormones involved in the stress response could explain variations in oxidative stress levels resulting from acute restraint-stress. For instance, adrenaline increases very rapidly during the first seconds to minutes after stress exposure, and has been shown to affect oxidative balance *in vitro*^58^.

Although restraint stress is a widely used method to measure the ability to mount an acute stress response in free-living animals, this stressor is not directly comparable to natural stressors (*e.g*. predator encounter). Future studies thus need to extend our evaluation of the oxidative stress response to more natural acute stressors (*e.g*. simulated predation event). This should enable a clear evaluation of the relevance of using restraint-stress as a methodological approach to study oxidative stress response in wild animals.

### Relationships between baseline GCs, mitochondrial function and oxidative stress

Although relationships between baseline CORT levels and mitochondrial function were only moderate (and non-significant after FDR correction), our results suggest that individuals with higher baseline CORT had slightly less efficient mitochondria at producing ATP for a given amount of O_2_ and substrate consumed (*i.e*. higher relative proton leak). This result might be surprising at a first glance, since one would expect individuals to optimize mitochondrial efficiency under harsh/demanding environmental conditions, which has been for instance demonstrated in king penguin chicks during their wintering fast^59^. Yet, decreasing mitochondrial efficiency is one known mechanism limiting ROS production, and thus oxidative stress^54,55^, even if the role of this mechanism is less established in birds than in other animals^60,61^. Decreasing mitochondrial efficiency in response to increased baseline CORT levels could be one useful mechanism to mitigate oxidative stress during chronic exposure to stressful stimuli.

Higher baseline CORT levels were not associated with higher oxidative damage levels, confirming our previous findings in this species^39^. Moreover, high CORT levels were associated to a low proportion of oxidized glutathione and low levels of protein oxidative damage, suggesting a potential beneficial effect of baseline CORT to maintain low levels of oxidative damage, although these effects were moderate (and non-significant after FDR correction). Besides changes in mitochondrial efficiency (see above), low levels of oxidative damage could be explained by an increase in antioxidant defences in response to elevated baseline CORT levels. Indeed, baseline CORT was positively correlated to the activity of the glutathione antioxidant system (*i.e*. total glutathione and GPx activity), suggesting that high baseline CORT could potentially trigger this pathway in our study species. The correlative nature of these results and the limitations inherent to the use of baseline CORT as an indicator of chronic stress prevent us however from reaching firm conclusions here. Indeed, baseline CORT could potentially reflect stress responsiveness rather than being a proxy of chronic stress exposure (*i.e*. as suggested by a positive correlation between baseline and T30 levels in our current dataset, Fig. S1), and more recently CORT has been suggested to reflect more metabolic demand than ‘stress’^62^. Future experimental studies should test if chronic stress and/or increased GC levels trigger an increase in endogenous antioxidants defences and a decrease in mitochondrial efficiency to prevent oxidative stress.

## Conclusion

In contrast to most previous studies^9,18,20–22^, our results show mostly no change or even a decrease in oxidative damage markers in relation to acute stress and high baseline or stress-induced GC levels. These findings are consistent with the hypothesis that animals dealing with repeated stressors in their environment, such as king penguins, may possess physiological mechanisms preventing oxidative damage to occur as a consequence of stress exposure. Interestingly, similar findings in captive house sparrows linking acute restraint-stress^19^ and experimental chronic elevation of GC hormones (*i.e*. CORT implant)^63^ to reduced oxidative damage have been recently documented. In penguins, part of this resistance to oxidative stress might be related to physiological adaptations to marine life and long-term fasting as recently shown in this species^64^. The role of mitochondrial uncoupling (*i.e*. decreased efficiency) as a potential mechanism of oxidative stress prevention during chronic stress exposure deserves further investigation, especially in the light of a recent study showing a reduced ROS production in response to CORT treatment despite no effect on mitochondrial bioenergetics in captive lizards^65^. Experimental manipulations of GC levels (*e.g*. via exogenous administration) and chronic stress (*e.g*. repeated simulated predation events) are now required to demonstrate causal effects of GCs and chronic stress on mitochondrial function and oxidative stress in wild animals.

## Supplementary information

ESM

## Data Availability

The dataset used in this manuscript is available at: 10.6084/m9.figshare.7042049.v1.
