# Supplementary material for: Oxidative stress and mitochondrial responses to stress exposure suggest that king penguins are naturally equipped to resist stress
Source: Sci Rep. 2019 Jun 12;9:8545. doi: 10.1038/s41598-019-44990-x (PMC6561961; doi:10.1038/s41598-019-44990-x)
Supplement: Supplementary file 1 — ESM [file 41598_2019_44990_MOESM1_ESM.pdf]

Electronic Supplementary Material (ESM)

Oxidative stress and mitochondrial responses to stress exposure suggest that king penguins are naturally equipped to resist stress

Antoine Stier, Quentin Schull, Pierre Bize, Emilie Lefol, Mark Haussmann, Damien Roussel, Jean-Patrice Robin, & Vincent A Viblanc

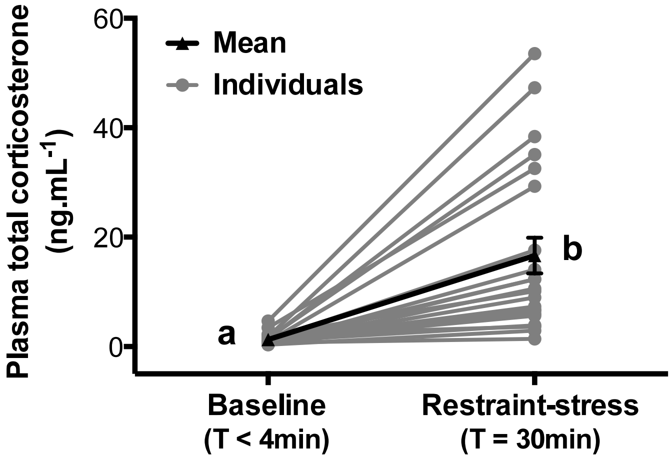

<u>Fig. S1</u>: **Plasma concentration of total corticosterone at baseline (< 4min of capture) and in response to an acute restraint-stress protocol (*i.e.* 30 min of standardized handling).** Individual responses are shown in grey and the mean population response ± SE is shown in black. Different letters indicate significant differences between the two sampling times (*i.e.* populations means) according to statistical model presented in the main text.

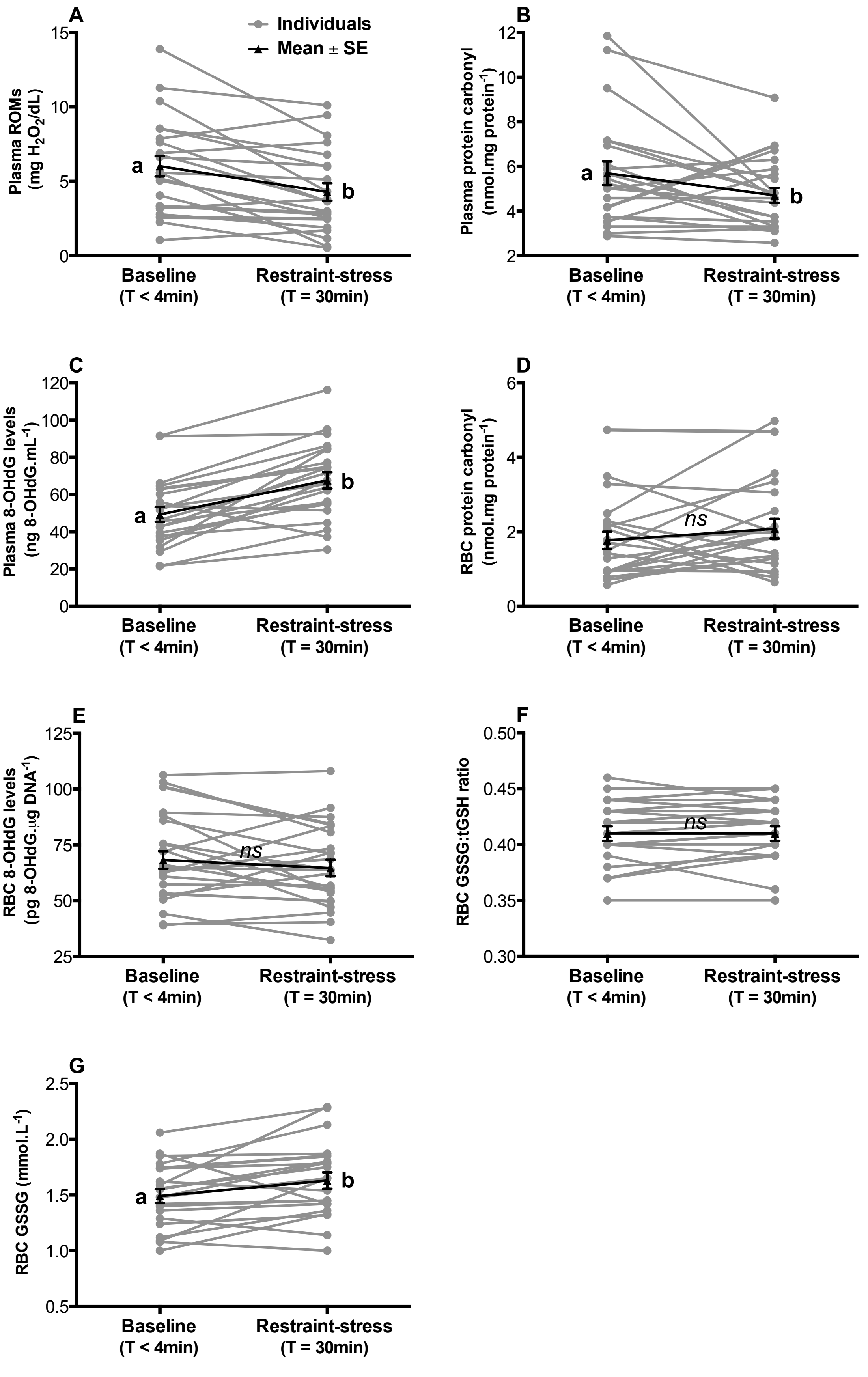

<u>Fig. S2</u>: **Oxidative damage markers at baseline (< 4min of capture) and in response to an acute restraint-stress protocol (*i.e.* 30 min of standardized handling). (A) Plasma ROMs, (B) Plasma protein carbonyl, (C) Plasma 8-OHdG, (D) RBC protein carbonyl, (E) RBC 8-OHdG, (F) RBC GSSG:tGSH ratio and (G) RBC GSSG.** The biological meaning of the different markers is explained in Table 1. Individual responses are shown in grey and the mean population response ± SE is shown in black. Different letters indicate significant differences between the two sampling times (*i.e.* populations means) according to statistical models presented Table S1.

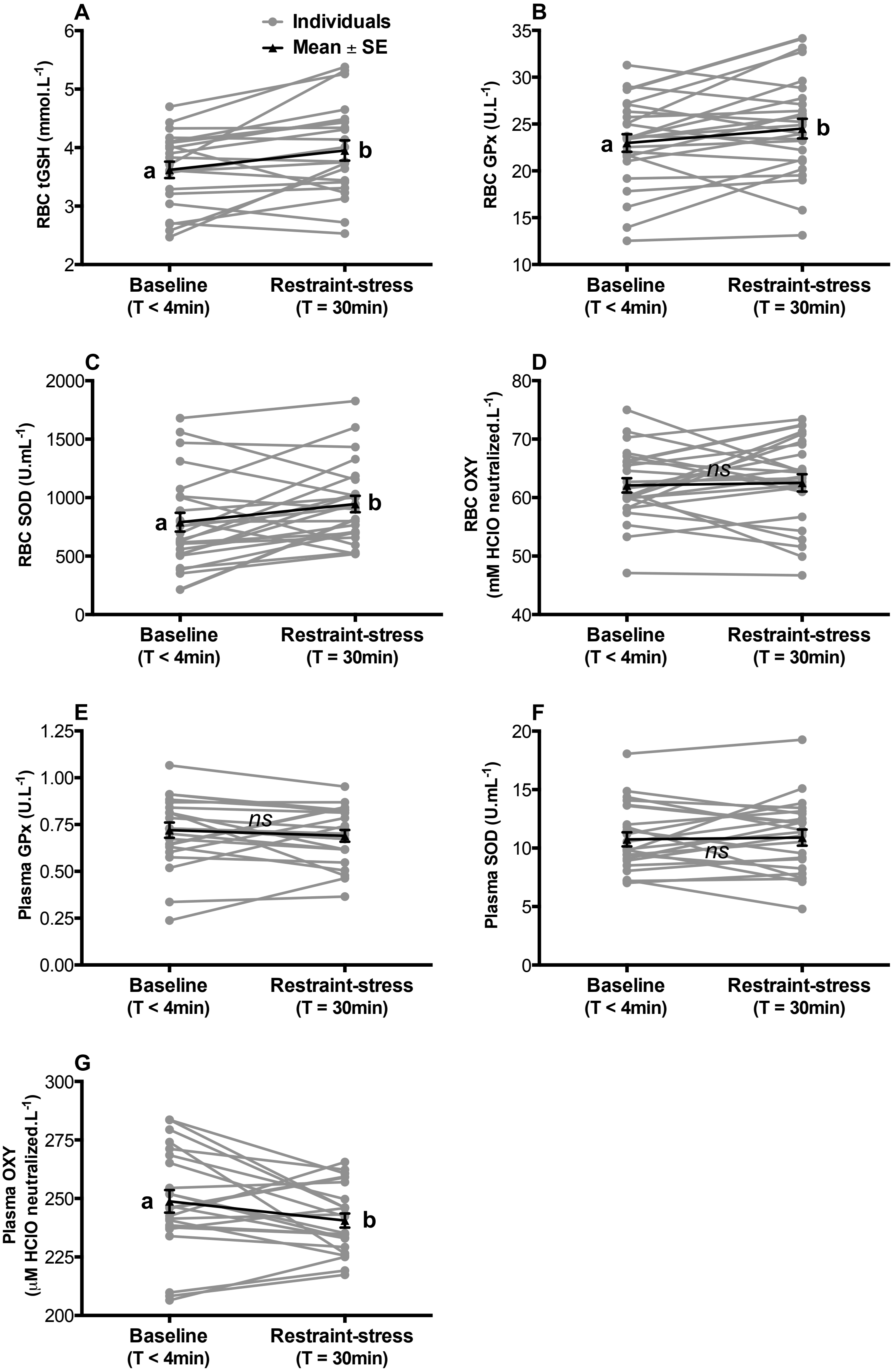

<u>Fig. S3</u>: **Antioxidant markers at baseline (< 4min of capture) and in response to an acute restraint-stress protocol (*i.e.* 30 min of standardized handling). (A) RBC tGSH, (B) RBC GPx, (C) RBC SOD, (D) RBC OXY, (E) Plasma GPx, (F) Plasma SOD and (G) Plasma OXY.** The biological meaning of the different markers is explained in Table 1. Individual responses are shown in grey and the mean population response ± SE is shown in black. Different letters indicate significant differences between the two sampling times (*i.e.* populations means) according to statistical models presented Table S1.

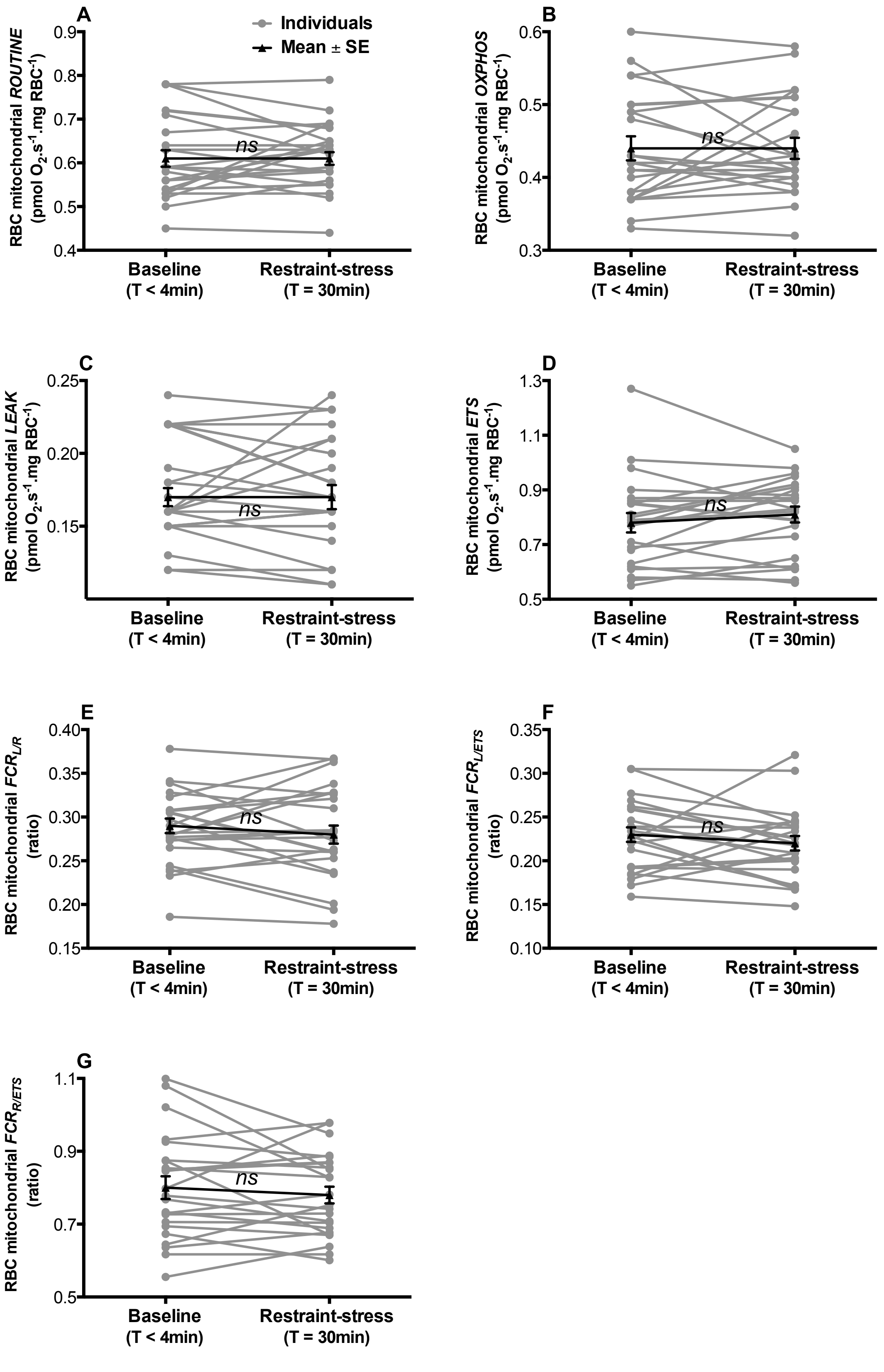

<u>Fig. S4</u>: **Mitochondrial respiration rates and flux control ratios at baseline (< 4min of capture) and in response to an acute restraint-stress protocol (*i.e.* 30 min of standardized handling). (A) *ROUTINE*, (B) *OXPHOS*, (C) *LEAK*, (D) *ETS*, (E) $FCR_{L/R}$, (F) $FCR_{L/ETS}$ and (G) $FCR_{R/ETS}$.** The biological meaning of the different markers is explained in Table 1. Individual responses are shown in grey and the mean population response ± SE is shown in black. Different letters indicate significant differences between the two sampling times (*i.e.* populations means) according to statistical models presented Table S1.

Table S1: **Summary of the statistical models (GEEs) testing the effects of acute restraint-stress exposure and sex on: (A) oxidative stress parameters and (B) mitochondrial parameters.** Significant parameters ($p \leq 0.05$) are reported in bold. Parameters remaining significant after false discovery rate (FDR) correction are underlined and marked with a *. Estimates are given for T30 samples and for females.

	**Acute restraint-stress**		**Sex**	
**A) Oxidative stress markers**	Estimate ± SE	p-value (χ^2)	Estimate ± SE	p-value (χ^2)
**<u>Plasma ROMs</u>**	**-1.73 ± 0.44**	**<u>< 0.001 (15.48)*</u>**	**3.64 ± 0.83**	**<u>< 0.001 (19.07)*</u>**
**Plasma protein carbonyl**	**-0.98 ± 0.48**	**0.039 (4.27)**	0.27 ± 0.69	0.69 (0.15)
**<u>Plasma 8-OHdG</u>**	**18.40 ± 3.13**	**<u>< 0.001 (34.50)*</u>**	7.62 ± 7.74	0.33 (0.97)
RBC protein carbonyl	0.31 ± 0.24	0.20 (1.65)	-0.02 ± 0.46	0.97 (0.00)
RBC 8-OHdG	-3.69 ± 2.93	0.21 (1.59)	**-14.08 ± 6.27**	**0.025 (5.04)**
**<u>RBC GSSG</u>**	**0.14 ± 0.05**	**<u>0.008 (7.09)*</u>**	0.02 ± 0.12	0.86 (0.03)
RBC GSSG/GSH	0.001 ± 0.003	0.68 (0.17)	0.010 ± 0.011	0.39 (0.75)
**<u>RBC total GSH</u>**	**0.33 ± 0.12**	**<u>0.006 (7.62)*</u>**	-0.07 ± 0.29	0.82 (0.05)
**RBC GPx**	**1.53 ± 0.71**	**0.031 (4.63)**	-0.01 ± 1.82	0.99 (0.00)
**<u>RBC SOD</u>**	**154.6 ± 61.8**	**<u>0.012 (6.26)*</u>**	-0.1 ± 134.0	0.99 (0.00)
RBC OXY	0.44 ± 1.28	0.73 (0.12)	-3.48 ± 2.25	0.12 (2.39)
Plasma GPx	-0.026 ± 0.028	0.35 (0.88)	0.097 ± 0.065	0.14 (2.19)
Plasma SOD	0.15 ± 0.49	0.76 (0.09)	**-2.73 ± 1.05**	**0.009 (6.80)**
**Plasma OXY**	**-8.23 ± 3.85**	**0.033 (4.57)**	3.40 ± 6.72	0.61 (0.26)
**B) Mitochondrial parameters**	Estimate ± SE	p-value (χ^2)	Estimate ± SE	p-value (χ^2)
ROUTINE	0.005 ± 0.014	0.70 (0.15)	0.027 ± 0.032	0.40 (0.71)
LEAK	0.001 ± 0.053	0.84 (0.04)	0.019 ± 0.013	0.15 (2.09)
OXPHOS	0.004 ± 0.012	0.72 (0.12)	0.007 ± 0.027	0.79 (0.07)
ETS	0.026 ± 0.021	0.23 (1.45)	0.039 ± 0.058	0.51 (0.43)
$FCR_{L/R}$	-0.001 ± 0.008	0.87 (0.03)	0.020 ± 0.018	0.26 (1.25)
$FCR_{L/ETS}$	-0.008 ± 0.008	0.36 (0.85)	0.018 ± 0.014	0.22 (1.54)
$FCR_{R/ETS}$	-0.023 ± 0.020	0.26 (1.29)	0.008 ± 0.049	0.87 (0.03)

Table S2: **Summary of the statistical models (GEEs) testing the relationships between the magnitude of the relative corticosterone acute stress response (Δ CORT) and the magnitude of the: (A) acute oxidative stress responses (Δ oxidative stress), and (B) acute mitochondrial responses (Δ mitochondria).** Significant parameters (p ≤ 0.05) are reported in bold, and parameters being marginally significant (p ≤ 0.10) are reported in italic. Parameters remaining significant after false discovery rate (FDR) correction are underlined and marked with a *. Estimates for sex are given for females.

	**Δ CORT**		**Sex**	
**A) Δ Oxidative stress markers**	Estimate ± SE	p-value (F)	Estimate ± SE	p-value (F)
Δ Plasma ROMs	0.049 ± 0.033	0.15 (2.26)	1.00 ± 0.95	0.30 (1.12)
Δ Plasma protein carbonyl	0.011 ± 0.038	0.77 (0.09)	-0.21 ± 1.09	0.85 (0.04)
Δ Plasma 8-OHdG	-0.041 ± 0.245	0.87 (0.03)	4.30 ± 7.07	0.55 (0.37)
ΔRBC protein carbonyl	0.006 ± 0.018	0.76 (0.10)	-0.331 ± 0.55	0.55 (0.36)
ΔRBC 8-OHdG	0.12 ± 0.24	0.61 (0.27)	9.88 ± 6.92	0.17 (2.04)
Δ RBC GSSG	0.002 ± 0.005	0.75 (0.10)	-0.03 ± 0.13	0.84 (0.04)
ΔRBC GSSG/GSH	0.000 ± 0.000	0.86 (0.035)	-0.008 ± 0.006	0.21 (1.75)
Δ RBC total GSH	0.003 ± 0.011	0.76 (0.10)	-0.02 ± 0.30	0.96 (0.00)
Δ RBC GPx	-0.004 ± 0.058	0.95 (0.10)	-1.37 ± 1.69	0.43 (0.66)
Δ RBC SOD	6.81 ± 4.99	0.19 (1.87)	-39.94 ± 144.13	0.79 (0.08)
**ΔRBC OXY**	**0.29 ± 0.08**	**0.003 (11.6)**	0.81 ± 2.45	0.74 (0.11)
ΔPlasma GPx	-0.001 ± 0.002	0.78 (0.08)	-0.049 ± 0.063	0.45 (0.60)
ΔPlasma SOD	0.013 ± 0.037	0.73 (0.12)	-1.00 ± 1.07	0.37 (0.86)
Δ Plasma OXY	-0.14 ± 0.28	0.62 (0.26)	-14.03 ± 8.20	0.10 (2.93)
**B) Δ Mitochondrial parameters**	Estimate ± SE	p-value (F)	Estimate ± SE	p-value (F)
ΔROUTINE	0.001 ± 0.033	0.58 (0.32)	-0.014 ± 0.032	0.67 (0.19)
**ΔLEAK**	**0.001 ± 0.000**	**0.050 (4.40)**	0.009 ± 0.012	0.48 (0.52)
ΔOXPHOS	0.000 ± 0.000	0.80 (0.07)	-0.023 ± 0.028	0.42 (0.68)
ΔETS	0.001 ± 0.002	0.62 (0.26)	0.031 ± 0.049	0.53 (0.41)
Δ$FCR_{L/R}$	*0.001 ± 0.001*	*0.09 (3.12)*	0.019 ± 0.017	0.28 (1.23)
Δ$FCR_{L/ETS}$	0.001 ± 0.001	0.31 (0.85)	0.000 ± 0.020	0.99 (0.00)
Δ$FCR_{R/ETS}$	0.000 ± 0.002	0.96 (0.00)	-0.063 ± 0.049	0.22 (1.65)

__Table S3__: **Summary of the statistical models (GLMs) testing the relationships between baseline corticosterone as a proxy of chronic stress exposure and (A) oxidative stress parameters at baseline or (B) mitochondrial parameters at baseline.** Sex was also included as a fixed factor in the models. Significant parameters (p ≤ 0.05) are reported in bold, and parameters being marginally significant (p ≤ 0.10) are reported in italic. Parameters remaining significant after false discovery rate (FDR) correction are underlined and marked with a *. Estimates for sex are given for females.

	**Baseline CORT**		**Sex**	
**A) Oxidative stress at baseline**	Estimate ± SE	p-value (F)	Estimate ± SE	p-value (F)
Plasma ROMs	-0.27 ± 0.62	0.67 (0.19)	**3.22 ± 1.27**	**0.021 (6.38)**
**Plasma protein carbonyl**	**-1.08 ± 0.51**	**0.046 (4.56)**	-0.19 ± 1.10	0.86 (0.03)
Plasma 8-OHdG	-1.76 ± 4.22	0.68 (0.17)	4.27 ± 8.67	0.63 (0.24)
RBC protein carbonyl	0.13 ± 0.25	0.61 (0.28)	0.26 ± 0.52	0.62 (0.26)
RBC 8-OhdG	-3.98 ± 3.26	0.24 (1.50)	**__-25.71 ± 6.68*__**	**0.001 (14.81)**
**RBC GSSG/GSH**	**-0.030 ± 0.011**	**0.017 (7.16)**	0.010 ± 0.012	0.42 (0.68)
RBC GSSG	0.16 ± 0.13	0.26 (1.39)	0.03 ± 0.15	0.85 (0.04)
**RBC total GSH**	**0.64 ± 0.26**	**0.030 (6.09)**	-0.05 ± 0.29	0.86 (0.03)
RBC GPx	*2.03 ± 0.99*	*0.055 (4.19)*	1.74 ± 2.03	0.40 (0.73)
RBC SOD	-108.47 ± 90.25	0.25 (1.44)	-37.95 ± 185.23	0.84 (0.04)
RBC OXY	-1.58 ± 1.33	0.25 (1.43)	-2.59 ± 2.73	0.35 (0.90)
Plasma GPx	-0.04 ± 0.04	0.39 (0.76)	0.10 ± 0.09	0.25 (1.42)
Plasma SOD	-0.84 ± 0.57	0.15 (2.22)	**-2.66 ± 1.16**	**0.034 (5.24)**
Plasma OXY	-7.25 ± 4.81	0.15 (2.28)	5.58 ± 9.86	0.58 (0.32)
**B) Mitochondrial parameters at baseline**	Estimate ± SE	p-value (F)	Estimate ± SE	p-value (F)
ROUTINE	-0.005 ± 0.022	0.82 (0.06)	0.028 ± 0.046	0.55 (0.37)
LEAK	0.010 ± 0.008	0.22 (1.61)	0.024 ± 0.016	0.15 (2.30)
OXPHOS	-0.015 ± 0.018	0.41 (0.71)	0.004 ± 0.037	0.92 (0.01)
ETS	-0.020 ± 0.040	0.62 (0.25)	0.020 ± 0.084	0.82 (0.05)
$FCR_{L/R}$	*0.018 ± 0.009*	*0.069 (3.73)*	0.028 ± 0.019	0.17 (2.05)
**$FCR_{L/ETS}$**	**0.021 ± 0.008**	**0.024 (6.10)**	*0.032 ± 0.017*	*0.084 (3.34)*
$FCR_{R/ETS}$	0.013 ± 0.035	0.71 (0.14)	0.034 ± 0.074	0.65 (0.21)
